# Influence of Wetting and Drying Cycles on Physical and Mechanical Behavior of Recycled Aggregate Concrete

**DOI:** 10.3390/ma13245675

**Published:** 2020-12-12

**Authors:** Caroline S. Rangel, Mayara Amario, Marco Pepe, Enzo Martinelli, Romildo D. Toledo Filho

**Affiliations:** 1 Department of Civil Engineering—COPPE, Federal University of Rio de Janeiro, 21941-972 Rio de Janeiro, Brazil; carolrangel@poli.ufrj.br (C.S.R.); mayara_amario@poli.ufrj.br (M.A.); toledo@coc.ufrj.br (R.D.T.F.); 2 Department of Civil Engineering, University of Salerno, 84084 Fisciano (SA), Italy; e.martinelli@unisa.it; 3 TESIS srl, 84084 Fisciano (SA), Italy

**Keywords:** recycled concrete aggregate, recycled aggregate concrete, durability, wet-dry cycles, mechanical properties, open porosity

## Abstract

Recently, concerns have been rising about the impact of increasing the depletion of natural resources and the relevant generation of construction and demolition waste, on the environment and economy. Therefore, several efforts have been made to promote sustainable efficiency in the construction industry and the use of recycled aggregates derived from concrete debris for new concrete mixtures (leading to so-called recycled aggregate concrete, RAC) is one of the most promising solutions. Unfortunately, there are still gaps in knowledge regarding the durability performances of RAC. In this study, we investigate durability of structural RAC subjected to wet-dry cycles. We analyze the results of an experimental campaign aimed at evaluating the degradation process induced by wetting and drying cycles on the key physical and mechanical properties of normal- and high-strength concrete, produced with coarse recycled concrete aggregates (RCAs) of different sizes and origins. On the basis of the results we propose a degradation law for wetting and drying cycles, which explicitly makes a possible correlation between the initial concrete porosity, directly related to the specific properties of the RCAs and the resulting level of damage obtained in RAC samples.

## 1. Introduction

The construction sector is one of the most environmentally impactful industrial activities in the world, as it requires a significant amount of natural resources and energy and generates an enormous volume of waste [1,2]. Therefore, nowadays, the reuse and recycling of construction and demolition waste (CDW) is a convenient solution to make this sector more “circular”. Although this poses many challenges, significant efforts have been made in relation to the recycling of concrete and other cement-based waste, such as the production of recycled concrete aggregates (RCAs) [3]. This has been motivated by the need to reduce landfilled CDW and by the growing scarcity of viable sources of virgin aggregates, mainly in regions close to metropolitan areas [4,5].

In a historical context, interest in RCAs began in the 1970s [6,7]. Since then, there has been a steady growth in scientific papers published in this area. Thomas et al. (2020) [8] pointed out that the use of RCAs was a highly relevant topic for the scientific community, coupled with an increased social need to preserve resources and to value waste. The importance of studying the characteristics of the RCAs is explained by the high percentage of volume that aggregates occupy in the concrete, and therefore their properties significantly influence the properties of the final concrete [9].

The main difference between RCA and ordinary aggregate is the presence of an aged mortar, the so-called attached mortar (AM), on the recycled aggregates [10]. This difference implies that RCAs present higher porosity, lower density, and higher water absorption capacity [11]. Thus, it is well known that water absorption potential is the main property that differentiates recycled aggregate from natural aggregate. The absorption of water occurs through the pore structure of the aggregate, allowing a greater absorption of fluid into the solid skeleton. Mortars, being more porous, absorb more water than natural aggregates, therefore, the mortar attached to the grains of the recycled aggregate causes this greater water absorption potential of the material [12].

Consequently, recycled aggregate concrete (RAC) could also present higher absorption and higher porosity in relation to conventional concrete produced with natural aggregates. This makes durability a problem, since aggressive agents find it easier to enter concrete [13,14,15,16]. In addition, the internal structures of natural concrete and RAC are different. In conventional concrete, there is only one type of interfacial transition zone (ITZ), which is between the natural aggregate and the cement paste. In recycled concrete, there are, in fact, two types of ITZs, i.e., one between the RCA and the new cement paste and another (inside the RCA) between the original aggregate and the attached mortar from the original concrete [17]. These ITZs are strongly related to the mechanical performance and durability of RACs, because the failure of concrete produced with RCA can occur in both ITZs [18].

The investigation of durability is certainly a very important aspect for the development and application of a new material. Durability can be defined as the ability of concrete to resist the actions of external agents without compromising the performance for which it was designed during its useful life. Therefore, the durability of isolated concrete and concrete structures depends on the proper selection of materials, mix-design, production and curing, and also depends on the interactions of the final material with its exposed environment [19]. Temperature, humidity, wind, and rain are the main agents of natural degradation of concrete in several environments. These factors interfere with the physical-mechanical behavior of concrete and the durability of structures [20].

Wu et al. [21] explained that the degradation processes that affected concrete, mostly, with the exception of mechanical damage, involved the flow of fluids through the porous structure of concrete. As a result, the durability of concrete depends largely on its ability to resist water penetration and other attacks. Thus, the relative humidity of the air and the incidence of rainfall are responsible for the availability of necessary water for degradation reactions to occur inside the concrete. In addition, Ibrahim et al. [22] pointed out that several problems found in concrete in fresh and hardened states were related to the exposure of the material to high temperatures throughout its useful life. Almusallam [19] reported that the development of mechanical strength and the porosity were the properties of hardened concrete most affected by temperature variation.

However, there is no single parameter to determine the durability of concrete structures, especially when subjected to the actions of potentially aggressive environments. It is possible to take into account durability issues through mechanical properties, such as compressive strength and elastic modulus. Most of the structures in service experience wetting and drying cycles and temperature variation and, as a result, concrete structures are rarely completely dry, fully saturated, or in constant thermal conditions [21]. To study the exposure to typical external conditions of the effects of rain, a simulation of this situation is carried out through tests of accelerated wetting and drying cycles.

The influence of wetting and drying cycles on concrete was analyzed by numerical resolutions in a study by Li et al. [23], and it was verified that the transport of moisture during drying was driven by evaporation and diffusion, while the transport of moisture during wetting was driven by absorption. The exposure of concrete to these situations produces some effects. Drying causes removal of water from the pores, stimulating shrinkage and micro cracking; wetting, despite causing water to recover from the microstructure, does not guarantee that the changes that occur in the drying phase will be reversed [21].

In the case of RCAs, according to Diagne et al. [24], the higher water absorption capacity of recycled aggregates combined with the seasonal variation of humidity and temperature could significantly influence the performance of the concrete. The authors carried out an experimental investigation on the use of RCA in concrete subjected to 0, 5, and 10 wetting-drying cycles (cycles of 1 h of saturation followed by 24 h of drying) and claimed that the durability of the concrete was strongly influenced by the wetting-drying cycles. In addition, the higher the percentage of RCA used, the faster the water drained through the concrete (due to the higher porosity of RCAs).

Observing the absorption results of RAC samples, Oliveira et al. [25] reported an increase in absorption as the amount of RCA in the samples increased, a fact that was expected due to the high absorption of recycled materials. An increase in the absorption of recycled concrete was also observed with an increase in the number of wet and dry cycles. It is worth noting that, for higher numbers of cycles, the presence of RCA accelerated the concrete degradation process.

Qi et al. [26] evaluated the degradation process of recycled concrete produced with different substitutions of coarse RCAs (30, 50, 70, and 100%) exposed to wetting and drying cycles, however, in sodium sulfate solution. Even though other factors should be considered in the case of exposure to sulfates, some comments from the authors were interesting for the present study. The authors stated that the higher absorption of the RCAs resulted in an obvious impact on the resistance of concrete against any type of attack, therefore, this property showed a direct relationship with the resistance of the recycled concrete to degradation processes. In addition, the authors explained that cracks were generated when the local stress was greater than the tensile strength of the concrete, which led to (visible) fragmentation of the surface of samples subjected to wet and dry cycles.

Although studies on the durability of concrete produced with RCAs have already been published, the main topics involving RAC subjected to wetting and drying cycles are mainly cases of exposure to sulfates and chlorides, and therefore little information on the exposure of recycled concrete to isolated wet and dry cycles can be found in the literature. In this context, there is still a knowledge gap about the influence of rain effects of typical tropical zones on the durability of RAC, in particular, in relation to processes of alternating cycles of wetting and drying together with temperature variations.

The present study reports on an experimental investigation aimed at analyzing the influence of the use of RCAs of different origins and sizes on wet-dry durability of normal (35 MPa) and high strength (60 MPa) concrete. Through a scientific mix-design technique for RACs previously developed by the authors [27], which considered the specific properties of the recycled aggregates, it was possible to carry out this evaluation on natural and recycled concrete of the same compressive strength. Thus, durability was assessed through the main physical and mechanical properties of ten structural concrete mixtures subjected to degradation processes by means of a different number of wet-dry cycles (0, 25 and 50 cycles).

## 2. Materials and Methods

### 2.1. Materials

#### 2.1.1. Cement and Superplasticizer

The cement used, in this study, was “high initial strength Portland cement” (Lafarge-Holcim, Rio de Janeiro, RJ, Brazil), labeled CPV-ARI, according to the National Brazilian Standard (NBR) 16697 [28], with the following main properties: specific gravity of 3181 kg/m^3^, 28-day compressive strength of 40.6 MPa, and actual packing density of 0.67.

The superplasticizer used in all mixes for workability control was a polycarboxilate superplasticizer, labeled “MC Powerflow 1180” (MC-Bauchemie, São Paulo, SP, Brazil), with the following main properties: specific gravity of 1070 kg/m^3^, solid concentration content of 35%, and saturation dosage of 1.5% (solids of SP based on the cement weight).

#### 2.1.2. Fine Aggregate: Natural

Natural quartz sand was used in this study as natural fine aggregate (NFA), with the following characterization: maximum grain size of 4.75 mm, specific gravity of 2447 kg/m^3^ [29], water absorption of 0.5% [30], and actual packing density of 0.70, 0.52 and 0.68 (3 size classes) [31].

#### 2.1.3. Coarse Aggregates: Natural and Recycled Concrete Aggregates (RCAs)

Granite type stones were used as the natural coarse aggregate (NCA) in the following two types of sizes: the first type was “NCA_C0” with a nominal diameter ranging from 4.75 to 9.5 mm (size named by “coarse aggregate 0”); the second type was “NCA_C1” with a nominal diameter ranging from 9.5 to 19 mm (size named by “coarse aggregate 1”).

**Figure 1:**
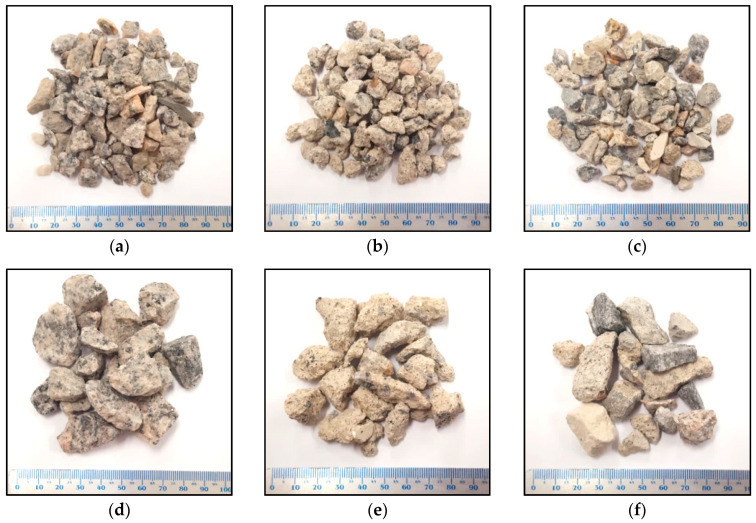
Natural coarse aggregates (NCAs) and recycled concrete aggregates (RCAs) used in this study. (**a**) NCA_C0; (**b**) RCA_L_C0; (**c**) RCA_D_C0; (**d**) NCA_C1; (**e**) RCA_L_C1; (**f**) RCA_D_C1 (adapted from [32]).

The recycled concrete aggregates (RCAs), employed herein, were obtained from the following two different sources [11]: concrete debris produced in laboratory (L-waste) and the debris of demolished concrete elements derived from a recycling plant (D-waste). In the production process of the RCAs, both concrete residues were broken into smaller pieces with a jaw crusher and, subsequently, the recycled aggregates were sieved in the two above-mentioned sizes, “coarse aggregate 0” and “coarse aggregate 1”, labeled “RCA_C0” and “RCA_C1”, respectively. Then, in the final stage, the RCAs were homogenized by longitudinal blending bed technique [17]. Figure 1 shows the two types of NCAs and the four types of RCAs used in this study.

In order to characterize both natural and recycled coarse aggregates, several tests were carried out and the main results are shown in Table 1, i.e., particles’ density and water absorption capacity [33], attached mortar content by thermal shock [11], “Los Angeles” abrasion [34], and actual packing density (3 size classes) [31].

The reported data showed that the properties of the aggregates depended mainly on the characteristics of the original concretes (L-waste and D-waste). Although the RCA particles obtained from the two sources presented similar properties for the fraction C0, for the class C1 this difference was more evident. In fact, since the L-waste had a maximum dimension of the original aggregate of 9.5 mm, generating aggregates of 19 mm led to greater amounts of attached mortar to the grains [11]. As expected, they also highlighted that the main properties of RCAs were directly related to the mortar content, i.e., the particles characterized by the greater amount of attached mortar also had a higher water absorption capacity and a lower density.

### 2.2. Methods

#### 2.2.1. Mixture Proportioning

In this study, a scientific mix design for the produced concrete mixtures was performed using the so-called compressive packing model (CPM) [31]. This method was originally developed for conventional structural concrete and has been recently extended for RACs [27,35]. The method is based on consideration of the specific characteristics of the constituent materials to meet the desired properties for concrete in both fresh and hardened state. In this study, these specific characteristics were the workability and the 28-day compressive strength. It is worth highlighting that adopting this methodology was one of the specific features of the present study, i.e., an individual mix design was developed for each mixture, and therefore the most common simple substitution of the natural aggregate by the recycled aggregate in the reference mixture was not carried out. For these reasons, the research, presented herein, was based on the assumption that the conclusion related to the durability of structural RAC performances should be performed by comparing concrete mixtures designed in an optimized way for controlling and achieving the desired concrete compressive strength which represented the parameter that, generally, classified the type of structural concrete mixture.

The optimization method was performed by maximum granular compactness of concrete, using the above-presented data of the actual packing density of the raw materials. The recycled materials were added to the mixture in a dry condition and the singular high absorption of the RCAs was considered during the definition of the mix proportioning. The absorption value of 50% of the total absorption obtained experimentally was used, since this value was previously shown (by Pepe et al. [36] and Amario et al. [27]) to be the amount that both fractions C0 and C1 absorbed during mixing.

The concrete mixtures were developed for the following two classes of compressive strength: normal strength with 35 MPa and high performance with 60 MPa. All mixtures were produced with 30% effective paste, and “effective paste” was the volume of free paste in the mixture (mixing water, cement, and superplasticizer) in relation to the total volume of concrete, in which the mixing (i.e., effective w_eff_) water did not take into account the absorption water of the aggregates. The aggregate proportion was fixed at 50 for sand, 25 for class C0, and 25% for class C1 in relation to the overall volume of aggregates (fine and coarse). For each class, five concrete mixtures were designed, varying the coarse aggregate, as follows:

(1) Reference with only natural aggregates (NCA_C0 and NCA_C1);
(2) 100% RCA_L_C0 in fraction C0 (RCA_L_C0 and NCA_C1);
(3) 100% of RCA_L_C1 in fraction C1 (NCA_C0 and RCA_L_C1);
(4) 100% RCA_D_C0 in fraction C0 (RCA_D_C0 and NCA_C1);
(5) 100% of RCA_D_C1 in fraction C1 (NCA_C0 and RCA_D_C1).

Table 2 shows the compositions of the concrete mixtures. The mixtures with only natural aggregates were named “CX-NAT”, with X indicating the strength class (35 or 60). The RCA mixtures were named “CX-Y-Z”, with X indicating the strength class (35 or 60), Y indicating the RCA source residue (L-waste or D-waste), and Z indicating the fraction used of RCA (C0 or C1).

From the composition of each concrete mixture and the content of attached mortar of each recycled aggregate, it was possible to calculate the percentage of total mortar volume (V_M,tot_) of each mixture (Table 2) by the sum of the volume of AM present in RCAs (V_AM_) and the new mortar volume (V_M,new_) given by the sum of sand, cement, water, and superplasticizer:

(1) $$V_{M,tot}=V_{AM}+V_{M,new}$$

(2) $$V_{AM}=\frac{W_{RCA,C0}}{\gamma_{RCA,C0}}\times AM_{RCA,C0}+\frac{W_{RCA,C1}}{\gamma_{RCA,C1}}\times AM_{RCA,C1}$$

(3) $$V_{M,new}=\frac{W_{eff,water}}{\gamma_{water}}+\frac{W_{CEM}}{\gamma_{CEM}}+\frac{W_{SP}}{\gamma_{SP}}+\frac{W_{sand}}{\gamma_{sand}}$$

where W_i_ represents the weight used for each fraction (from Table 2) and *γ*_i_ is the corresponding density (from Table 1).

#### 2.2.2. Two-Stage Mixing Approach (TSMA) of RAC

As already mentioned, all aggregates were added in dry condition to the mixture. The same mixing procedure was adopted for both normal and high strength concretes. Due to the high water absorption capacity of RCAs, a specific methodology for the mixing process was adopted, the well-known two-stage mixing approach (TSMA) developed and improved for cases of RAC by Tam et al. [37] and Tam and Tam [38,39], in which the total water was divided into two equal parts, and the parts were added at different times of the mixing.

The mixing procedure was performed in the following order: Initially, homogenization of all coarse and fine aggregates by mixing for 1 min; followed by the addition of half the total amount of water and mixing for 1 min for better absorption by RCAs; then, the cement was added and mixed for 1 min with the aggregates; finally, the rest of the water (second half) and the superplasticizer were added and mixed for 8 min (superplasticizer action time), concluding the concrete mixing process. The casting of the concrete was carried out in two layers, followed by mechanical compaction for 30 s. The samples were demolded after 24 h and the cure was carried out in a wet chamber with relative humidity of 100% and temperature of 21 °C.

#### 2.2.3. Wet-Dry Cycles Degradation Procedure

**Figure 2:**
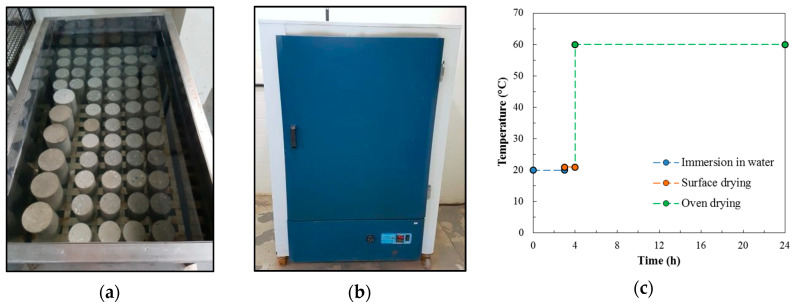
Wet-dry degradation procedure. (**a**) Wetting phase; (**b**) Drying phase; (**c**) One wet-dry cycle.

The degradation process by wetting and drying cycles started at the age of 28 days. Cylindrical samples, 75 mm in diameter and 150 mm in height, were used. Initially, the samples were saturated in water for 48 h, and then the cycles started. The durability test of concrete samples subjected to accelerated wetting and drying cycles was based on NBR 13554 [40] and ASTM D559 [41], and was performed manually and with the use of a water tank (Figure 2a) and a laboratory oven (Figure 2b). Each cycle consisted of the following three stages (as shown in Figure 2c): immersion in water at 20 °C for 3 h; superficial drying at 21 °C for 1 h; and, finally, oven-drying at 60 °C for 20 h. The total time of each cycle was 24 h and samples were submitted to 0, 25, and 50 wet-dry cycles.

After the degradation process, the concrete samples were characterized by determining their relevant mechanical properties, such as compressive strength, elastic modulus, tensile strength, and mass loss. The weighing of the samples was carried out in order to follow the mass variation. The weight loss test was performed on three samples of each mixture for each level of wet-dry degradation. The samples were weighed in an oven-dried condition (after mass consistency) before the degradation process. After 25 cycles, the same samples were weighed again in an oven-dried condition. The same procedure was performed for the samples of 50 wet-dry cycles. The calculated mass loss is the average of the percentage differences between the initial mass and the mass after degradation of each sample. It is worth mentioning that, for all mixtures, non-degraded samples were kept protected in a wet chamber (21 °C temperature and 100% humidity) and tested as reference at the same age as the samples degraded by 25 and 50 cycles.

#### 2.2.4. Testing Procedures

The characterization of all mixtures was carried out through the tests detailed in Table 3.

## 3. Results and Discussion

### 3.1. Workability, Physical, and Mechanical Properties of RAC

This section presents the results of the experimental characterization of the ten concrete mixtures produced in this study. Table 4 shows workability (slump), physical properties at 28 days (water absorption, void index, and specific gravity), and mechanical properties at 28 days (compressive strength, elastic modulus, and tensile strength).

Many authors have stated that the presence of RCA caused a reduction in the workability of concrete, caused mainly by the higher absorption capacity of these materials [46,47]. However, all concrete mixtures, in this study, showed satisfactory slump values of 180 ± 20 mm, which allowed excellent casting of concrete structures. The authors have reinforced that it was possible to produce RACs with workability similar to conventional natural concrete due to the individual specific mix design methodology of each mixture adopted in this study, in which a testing step was performed to guarantee the desired workability performance of each concrete mixture.

The results of physical properties show that recycled concrete has higher total water absorption (i.e., concrete open porosity, w_open_), higher void index, and lower specific gravity as compared with natural concrete. These results are consistent with the conclusions that have been obtained in several studies available in the literature [46,48].

Particularly, regarding water absorption, the high-performance class showed lower absorption than the normal strength class and, in both classes, the mixture with the lowest value was the one with 100% natural aggregates, as expected. In both strength classes, the mixture with the highest absorption capacity was that produced with the recycled aggregate RCA_L_C1, with an increase in absorption of 30% for C35 and 72% for C60, in relation to the absorption of the corresponding natural concretes. Therefore, the incorporation of RCAs proportionally causes a greater variation in the absorption of high-performance concretes.

Similar to absorption, the results for void index and specific gravity also follow the expected, i.e., concrete mixtures with 100% NCA are the ones that present the lowest void index values and the highest specific gravity values in both classes. The mixtures with 100% RCA_L_C1 have the highest proportional increase of 23% for C35 and 59% for C60, in relation to the void index of natural concretes. As for density, the highest difference in relation to natural concrete was obtained by the mixtures C35-L-C1 and C60-L-C1 with a decrease of 3.6% and 3.0%, respectively, in relation to the specific gravity of the corresponding conventional mixtures.

**Figure 3:**
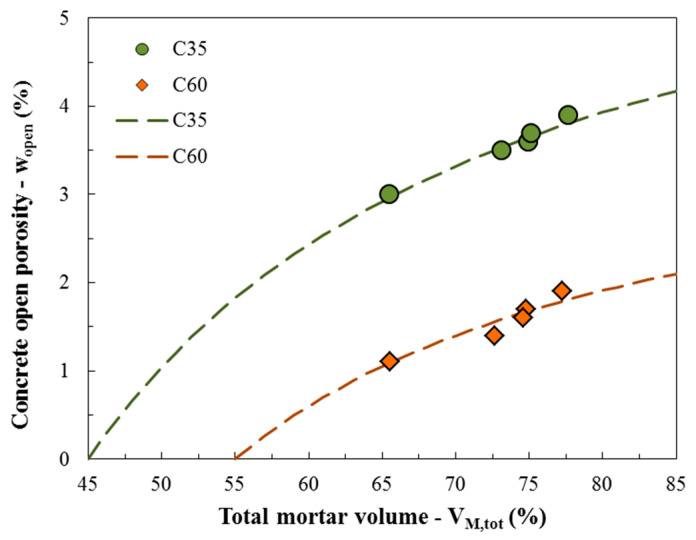
Relation between total mortar volume (V_M,tot_) and concrete open porosity (w_open_).

Meanwhile, from the percentage of total mortar volume of each mixture (as explained above, the sum of the mortar present in the RCAs with the new mortar of the mixture), the following relationship is shown in Figure 3: the higher the total mortar volume (V_M,tot_), the higher the open porosity of concrete (w_open_) (i.e., absorption). This is explained by the fact that the open porosity of concrete also depends on the raw materials employed within the mixture, and, in the specific case of RACs, it is influenced by the presence of the porous attached mortar inside or around the RCAs particles. Thus, the strong connection between these two parameters, that is, the trend of the increasing curves in Figure 3, can be described by the following proposed equation, where k_1_, k_2_, and V_M,0_ represent numerical parameters calibrated for both normal and high strength classes:

(4) $$w_{open}=\frac{k_{1}\times \left(V_{M,tot}-V_{M,0}\right)}{k_{2}+\left(V_{M,tot}-V_{M,0}\right)}$$

where k_1_ was calibrated with 7.3 for C35 and 4.2 for C60, k_2_ with 30.0 for both classes, and V_M,0_ with 45.0 for C35 and 55.0 for C60.

The results obtained from compressive strength at 28 days (f_c,28_) prove that all recycled concretes obtained similar values to natural concretes for the studied classes, according to the initial proposal of this study, that is, the premise to understand the influence of the presence of RCAs in concretes of the same strength subjected to degradation by repetitive wet-dry cycles. In fact, the maximum difference between the expected compressive strength and the experimentally obtained compressive strength was 4% for both normal strength and high-performance mixtures as compared with the predicted values of 35 and 60 MPa. These results reinforce the excellent capacity of the specific mix design methodology described (based on [31]) to predict the compressive strength of recycled concrete of different strength classes, provided that the data of the RCAs used are correctly provided, as well as the adequate consideration of their absorption capacities during mixing (50% of the total 24 h absorption).

For elastic modulus at 28 days (E_c,28_), as compared with the values obtained for concretes with 100% NCA, there was a maximum variation of 1.8% decrease to 3.7% increase in RACs of C35 (C35-D-C1 and C35-L-C0, respectively), and a maximum variation of 1.3% to 6.5% increase in RACs of C60 (C60-D-C1 and C60-D-C0, respectively). Finally, the results of splitting tensile strength at 28 days (f_t,28_) of C35 showed a variation of 3% decrease to a 7% increase in RACs in relation to the natural mixture (C35-D-C1 and both C35-L-C1 and C35-D-C0, respectively). The results of C60 expressed a maximum increase of 12% (from both C60-L-C1 and C60-D-C0) as compared with the result of the natural concrete. Therefore, in the high strength class, all RACs obtained a better result than the natural mixture for these two main mechanical properties.

It is worthwhile to highlight that the maximum scatter registered in the abovementioned results ranged between 1% and 12% and, this variation was the typical scatter also observed for ordinary concrete. Moreover, the variations observed in this case can also be explained by the following aspect: (i) the more porous structure (e.g., looking at the lower density presented in Table 1) of the recycled particles; (ii) the different mixture composition (i.e., amount of cement and effective water-to-cement ratio) characterizing the RAC mixtures; and (iii) the different ITZ characteristics present in RAC. In fact, on the one hand, the presence of a lower dense aggregates tends to reduce the overall elastic modulus of the composite, while, on the other hand, the lower effective water-to-cement ratio mitigates this phenomenon and, for this reason the maximum registered variation is lower than 4%. Regarding the tensile splitting strength, the higher values observed for RAC mixtures can be associated, also in this case to the lower effective water-to-cement ratio but also to the presence of a different ITZs which are characterized by a rougher surface and, consequently, can improve the mechanical response under tensile loads.

### 3.2. Physical and Mechanical Properties of RAC Subjected to Wet-Dry Cycles

The nomenclature “WD” was adopted from the expression “wet-dry”. The degraded samples were named by the number of wet-dry cycles that they were submitted, 25 and 50 cycles, represented by “WD25” and “WD50”, respectively. The reference samples were not degraded, and they were tested at the same age as the degraded ones, with the abbreviations “REF(WD25)” and “REF(WD50)”, according to the samples to which they serve as reference.

The physical and mechanical degradation of concrete subjected to wetting and drying cycles can be associated with the different hygrometric profile which is generated within the concrete element. As a matter of principle, during the wetting phase, the open pores are filled by water during drying, depending on the concrete size and the duration of each cycle, only the cortical area of the element is able to be fully dried. This phenomenon promotes the shrinkage of the external layers while the internal core (more humid than the external surface) locks the volume change [49]. This results in the creation of microcracks in the concrete elements and the fatigue effect amplify this phenomenon. Then, at the macro level, this is observed with the reduction of the physical and mechanical properties.

#### 3.2.1. Mass Loss

**Figure 4:**
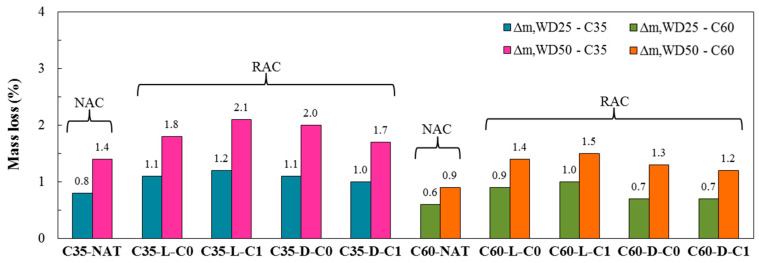
Mass loss after wet-dry degradation.

The mass loss results for natural aggregate concrete (NAC) and RAC after 25 and 50 wet-dry cycles are shown in Figure 4. The normal strength class presented larger mass losses than the high-performance class for both 25 and 50 cycles. This fact is associated with the higher porosity of C35, which allows a greater flow of water through its internal structure in the wetting stage, together with a lower resistance of its mortar to the shrinkage that occurs in the drying stage, causing micro cracking in concrete. This behavior is in agreement with that explained by Wu et al. [21].

In both strength classes, the mass loss rates do not show significant differences with an increased number of cycles. In general, the change in the mass of concrete (natural and RAC) occurs due to mortar fragmentation during wet-dry cycles. In the drying stage, a pressure arises due to the shrinkage of the concrete by the evaporation of the internal water, thus, the concrete becomes weaker and fragmented with the creation and evolution of micro cracks. This aspect is also confirmed by the fact that during the wet-dry tests there were small components of the concrete in the water tank. Consequently, the mass of the samples decreases. With the repetition of the cycles, this process repeats, causing more and more reductions in the mass. However, as shown in the results, this occurs at a (approximately) constant rate of change.

Finally, in both C35 and C60, mixtures produced with the RCA of class size C1 from laboratory waste (C35-L-C1 and C60-L-C1) stand out for higher mass loss as compared with other mixtures, while natural mixtures (C35-NAT and C60-NAT) have the lowest mass loss results. Therefore, with the joint action of wetting and drying processes, the results show that these interactions modify the properties and the microstructure of RACs more clearly than natural concretes. This fact can be explained as follows: Natural concrete presents only the new mortar in concrete undergoing this aggressive process, while RACs undergo these processes in the new mortar, and also in the old mortar attached to the grains of RCAs, consequently RACs suffer further damage.

#### 3.2.2. Compressive Stress-Strain Behavior

**Figure 5:**
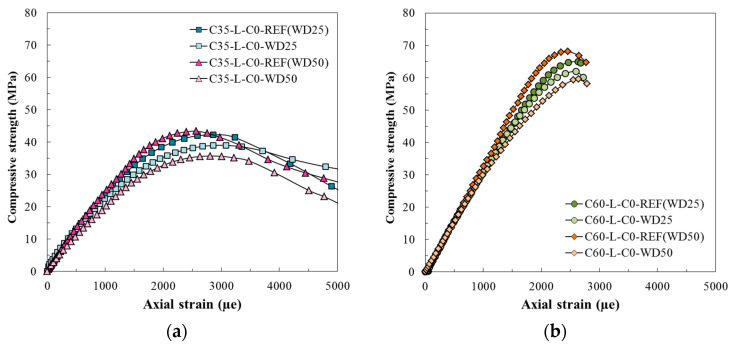
Typical compressive behavior of reference and after wet-dry degradation. (**a**) C35; (**b**) C60.

The typical stress-strain responses obtained in compressive strength tests of normal and high strength classes in the wet-dry degradation study of RACs are presented in Figure 5. For C35, all concrete mixtures showed a slight variation in compressive behavior after the first 25 degradation cycles, and this difference was identified by comparing the stress-strain curves obtained for REF(WD25) and WD25 (Figure 5a). The same occurred when comparing REF(WD50) with the curves of 50-cycles degraded concretes WD50. Specifically, the curves obtained after degradation have lower compressive strength and higher strain peak than their respective references. For C60, the same comments can be made, since the curves WD25 and WD50 present lower compressive capacity than their references, REF(WD25) and REF(WD50) (Figure 5b), that is, in the high strength class, the cycles also cause a decrease in compressive strength and an increase in strain peak.

**Figure 6:**
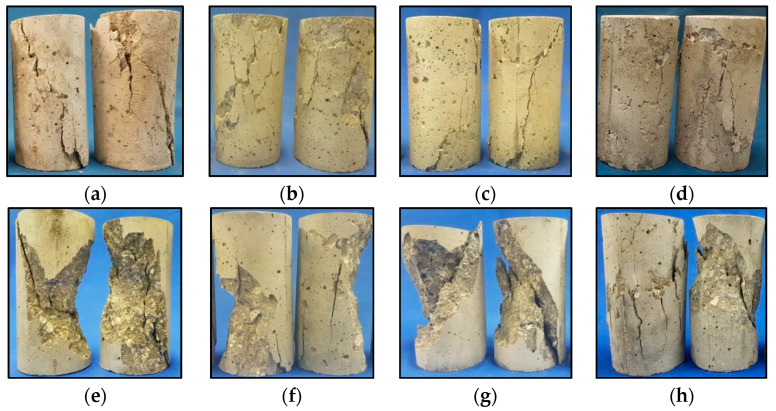
Typical rupture of C35 and C60 in the compressive strength test in the wet-dry durability study. (**a**) C35-REF(WD25); (**b**) C35-WD25; (**c**) C35-REF(WD50); (**d**) C35-WD50; (**e**) C60-REF(WD25); (**f**) C60-WD25; (**g**) C60-REF(WD50); (**h**) C60-WD50.

Samples of the typical rupture that occurred in each class in the compressive tests after wetting-drying degradation are shown in Figure 6. Typical rupture of reference samples of C35 consisted of well-defined diagonal cracks (REF(WD25) in Figure 6a and REF(WD50) in Figure 6c), similar to what was observed in the 28-day tests, however, cracks appeared to be less coordinated in the degraded samples, without forming such a clear diagonal (WD25 in Figure 6b and WD50 in Figure 6d). C60 reference samples exhibited a typical explosive rupture but still with a clear diagonal formation (REF(WD25) in Figure 6e and REF(WD50) in Figure 6g), similar to what was observed by the authors in the 28-day samples, and the degraded samples of C60 showed a crack pattern very similar to that of the reference samples, i.e., explosive rupture, but with a less evident diagonal, being possible to see cracks in other directions as well (WD25 in Figure 6f and WD50 in Figure 6h).

This variation in the cracking pattern of the samples after wet-dry cycles can be explained by the fragmentation of the mortar and the increase in the volume of pores, previously described. This modification in the internal structure of the concrete, caused by the degradation process, can result in a weaker “line” in the concrete (different from the typical main diagonal), which becomes the “path” of the cracking process during the application of stress in the compressive test.

**Figure 7:**
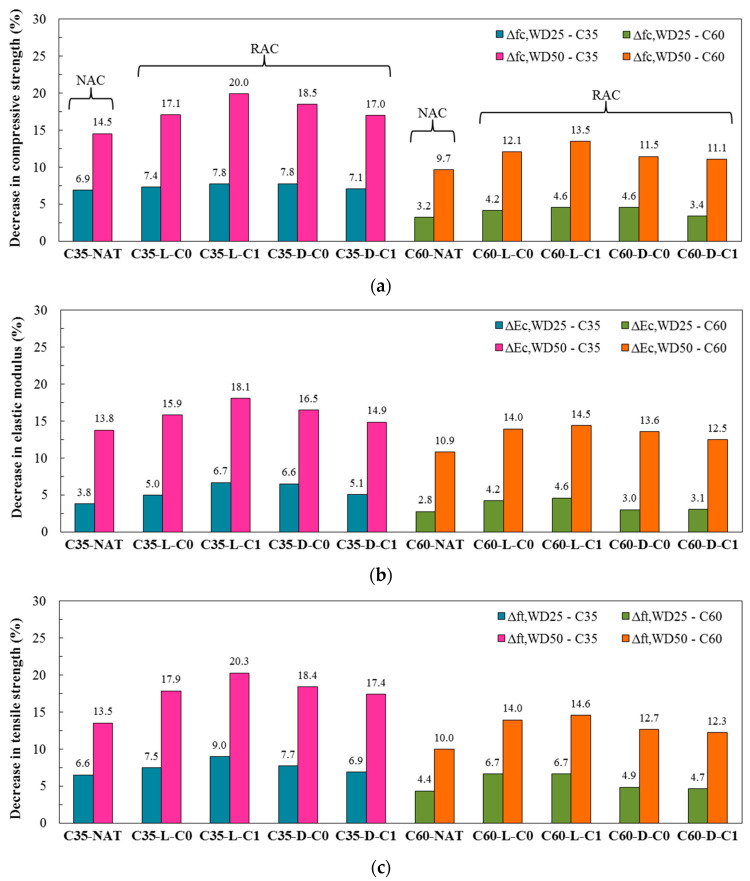
Decrease in mechanical properties after wet-dry degradation. (**a**) Compressive strength; (**b**) Elastic modulus; (**c**) Tensile strength.

Table 5 presents the results for the following three main mechanical properties of the concrete samples degraded by 25 and 50 wet-dry cycles (WD) and their respective control reference samples: compressive strength, elastic modulus, and tensile strength. Still, the percentage decreases of the degraded samples in relation to their reference samples are illustrated in Figure 7.

The results confirmed that all samples from both classes suffered a loss in compressive strength when subjected to repetitive wetting and drying cycles. The normal strength class reached degradation percentage decreases from 6.9% to 7.8% at the end of 25 cycles and from 14.5% to 20% after 50 cycles (Figure 7a). For lower numbers of cycles (0–25), the differences in behavior of the five mixtures were almost imperceptible (similar decreases in resistance). However, as the cycles increased (25–50), the performances of the mixtures began to differentiate. C35-L-C1 suffered the greatest impact on behavior (highest percentage decrease) and C35-NAT had the lowest impact on behavior (lowest percentage decrease). It can be noted that, for RACs, the drop rate increased with an increased number of cycles. This could be expected for all concrete samples, as the more degraded the samples, the lower resistance to subsequent cycles would be expected, although the conventional concrete showed similar rates for the two studied stages, 0–25 and 25–50 cycles. Therefore, it can be emphasized that for larger numbers of cycles, it is evident that the capacity of the natural mixture to resist degradation by wetting-drying cycles is greater than the RAC mixtures.

The percentage decrease in compressive strength was higher for the normal strength class than for the high-performance class for both two levels of cycles. This fact was expected due to the previously explained mass loss, i.e., it was related to the higher porosity (associated with greater water penetration in the wetting phase) and the lower resistance (associated with the lower capacity to withstand the stresses generated by the shrinkage in the drying phase) of C35. For C60, the compressive strength percentage decreases ranged from 3.2% to 4.6% after 25 wet-dry cycles and from 9.7% to 13.5% after 50 wet-dry cycles (Figure 7a). The same comments regarding C35 can be considered for C60, that is, the mixture with the best durability performance to higher degrees of wet-dry degradation is the natural concrete C60-NAT.

For C35, the values of elastic modulus decrease of samples degraded by 25 wet-dry cycles ranged from 3.8% to 6.7%, and, after 50 cycles, the values increased for 13.8% to 18.1% as compared with control samples (Figure 7b). For this particular property, the drop rate for the second phase of cycles (25–50) was higher than for the first phase (0–25) for all mixtures, indicating that the more degraded the sample the more the modulus was impacted by new wetting-drying cycles. The natural concrete C35-NAT presented the best behavior for both 25 and 50 wet-dry cycles, standing out as compared with RACs. The high strength class had lower degradation decreases of elastic modulus than the normal strength class, and it also related to the lower porosity and higher mortar resistance of C60 (already explained). The reductions of C60 were only 2.8% to 4.6%, after 25 cycles, but reached 10.9% to 14.5% when subjected to 50 wetting-drying cycles (Figure 7b). Again, the drop rates were higher for the second phase of cycles (25–50). Nevertheless, following the pattern observed for C35, the results obtained for C60 showed that the high strength natural concrete also stood out with the best resistance in relation to elastic modulus for higher levels of wetting and drying degradation.

#### 3.2.3. Tensile Strength

For the normal strength class, tensile strength values showed a decrease of 6.6% to 9.0% for samples degraded by 25 cycles, and a decrease of 17.4% to 20.3% for RACs, and 13.5% for natural concrete after 50 cycles (Figure 7c). Therefore, C35-NAT was the one that showed an evident better behavior when subjected to a higher number of degradation cycles. The drop rates of tensile strength for the two cycle phases (0–25 and 25–50) were very similar, with no significant increase in the second phase, different from what happened to compressive strength and elastic modulus. C60 showed lower percentage losses than C35 after cycles. For C60, the tensile strength percentage loss was between 4.4% and 6.7% for 25 cycles and it increased to 10% to 14.6% after 50 cycles. At the end of 50 cycles, C60-L-C1 had the highest percentage impact on this property and C60-NAT had the lowest.

## 4. Wet-Dry Degradation Law for RAC

With the experimental results obtained for the properties discussed above, a generalized degradation law is proposed for RAC subjected to wet-dry cycles. The law represents the relationships between an initial property of the intact concrete, in this case, the concrete open porosity (w_open_), and the variations obtained in the main mechanical properties of concrete after wet-dry degradation, that is, compressive strength (f_c_), elasticity modulus (E_c_), and tensile strength (f_t_). The three proposed relationships are described in the following equations:

(5) $$\frac{f_{c,WD}}{f_{c,REF}}=\frac{1}{1+a\times {\left(w_{open}\right)}^{2}}$$

(6) $$\frac{E_{c,WD}}{E_{c,REF}}=\frac{1}{1+b\times {\left(w_{open}\right)}^{2}}$$

(7) $$\frac{f_{t,WD}}{f_{t,REF}}=\frac{1}{1+c\times {\left(w_{open}\right)}^{2}}$$

where a, b, and c are calibrated parameters in relation to each of the properties, that is, compressive strength, elastic modulus, and tensile strength, respectively.

**Figure 8:**
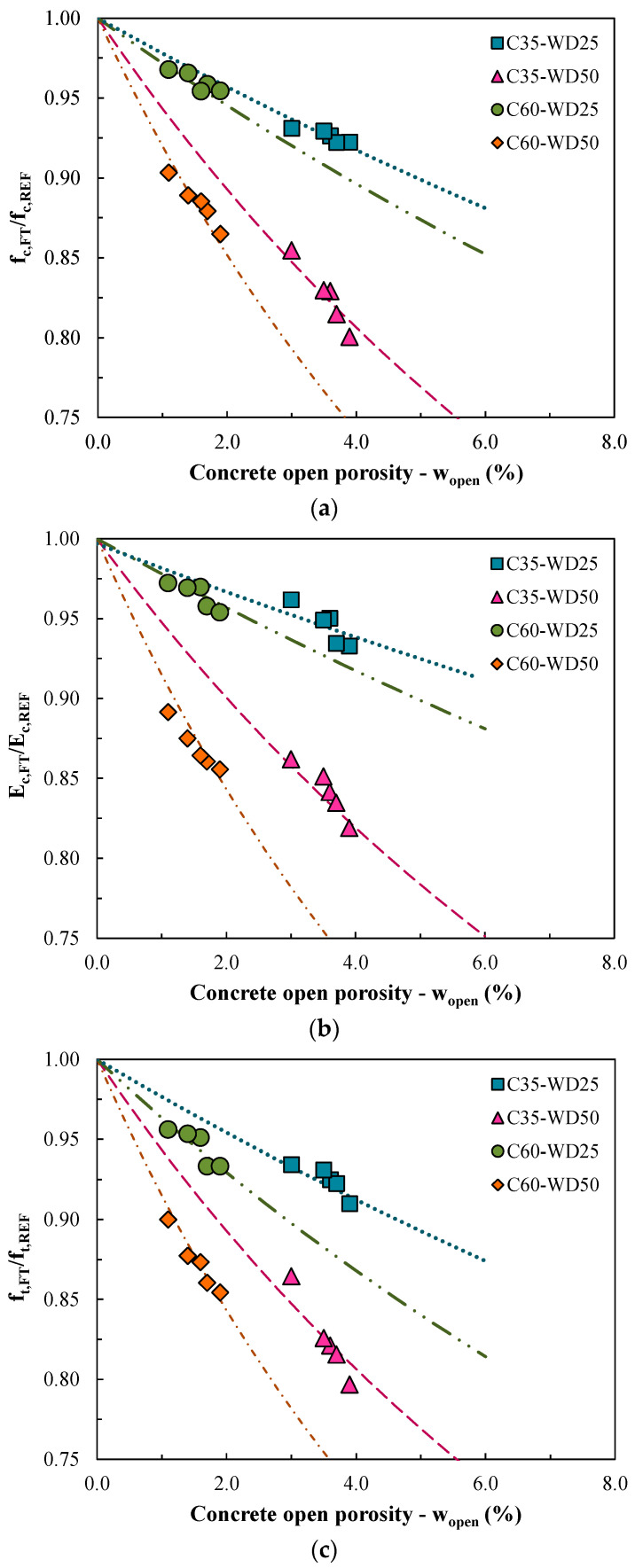
Relation between concrete open porosity and mechanical properties in the wet-dry durability study. (**a**) Compressive strength; (**b**) Elastic modulus; (**c**) Tensile strength.

In particular, the calibrated degradation-law curves proposed are shown in Figure 8 with their respective parameters a, b, and c (see Table 6), along with the experimental results of the wet-dry durability study. The equations express that the relationship between each mechanical property after the cycles and its reference is a function of the initial open porosity.

It is worthhile to mention that the proposed equations are based on a theoretical approach recently presented by the authors in a companion study devoted to analyzing the behavior of RAC mixtures subjected to freeze-thaw cycles [32].

This behavior clearly expresses that open porosity is directly related to wet-dry degradation of concrete. In fact, in cases where open porosity would theoretically be equal to zero (i.e., the extreme case of impermeability), there would be no degradation of concrete, since the water would not flow within the concrete structure and it would not cause damage to the material.

In all graphs, the following can be observed: On the one hand, the points above are the results for 25 wet-dry cycles (WD25) of the ten concrete mixtures and they indicate that the influence of this degradation level is less relevant; on the other hand, the observation of the lower points, which represent 50 wet-dry cycles (WD50), highlights two very evident trend lines, one for each strength class, where the right trend line represents C35 (higher porosity) and the left trend line represents C60 (lower porosity). Thus, two numbers (one for each resistance class) were calibrated for parameter a for compressive strength (Figure 8a), followed by the calibration of the elastic modulus trend through parameter b also by two numbers (Figure 8b) and, finally, the calibration of the parameter c was performed in the same way for tensile strength (Figure 8c).

In the three graphics, in addition, the points more to the left of each of the four groups of points refer to the mixtures of natural concrete (C35-NAT and C60-NAT), since these concretes have lower porosity in each class, because they have a lower total mortar volume than RACs (as already explained in Figure 3). It can be observed that although the behavior of RACs does not stand out for up to 25 cycles, the presence of a porous mortar adhered to the grains causes the concretes produced with RCAs to present greater decreases in their mechanical properties after 50 cycles than natural concretes. This fact is explained by the greater number of ITZs of recycled concrete, which represent a greater number of regions sensitive to degradation than natural concrete.

The shape of the curves shows that the graph of the high-performance class is more vertical for all three properties, which indicates that for a smaller variation in concrete porosity (*x*-axis) there is a greater variation in durability capacity (*y*-axis) than for the normal strength class. This confirms that the presence of a more porous aggregate causes a greater impact on the wet-dry resistance of C60. In other words, as the porosity of the aggregate is directly related to the attached mortar content, the durability of C60 is more sensitive to the presence of AM than C35.

This statement is also confirmed by the higher values of parameters a, b, and c for mixtures of C60 class in Figure 8. Finally, it is worth mentioning that no influence of the aggregate grain size was identified on the behavior of the concrete in any of the graphs, that is, the behaviors studied do not depend on the size class of the RCA itself, but on the consequence that this recycled material causes in the absorption of the new produced concrete.

In summary, the proposed wet-dry degradation law for RAC describes that the decrease in mechanical performance caused by wet-dry cycles is related to the initial open porosity of the concrete (Figure 8), which is related to the total mortar volume in the mixture (Figure 3), which is finally related to the mortar content of the RCAs (Equations (1)–(3)). Thus, the durability of RACs to wet-dry processes is strongly dependent on the main property of recycled aggregates, i.e., the attached mortar. However, if the wet-dry durability of the concrete depends on the initial porosity of the concrete, a viable solution that would allow its use in these extreme conditions of degradation would be the control of this physical property (i.e., open porosity) in the mix-design phase. That is, it would be possible to use RACs in regions of high temperature and humidity variation by controlling the open porosity of this sustainable material.

## 5. Conclusions

This study reports the results of an experimental study on the physical and mechanical performance of structural concrete made with either natural or recycled aggregates and subjected to degradation processes similar to environmental conditions of temperature and humidity variation. Specifically, it summarizes the results of an experimental investigation aimed at analyzing one of the aspects related to the durability of normal and high strength concretes produced with different types of RCAs, after being subjected to different levels of wetting-drying cycles. Particularly, the following considerations can be highlighted:

- All concrete mixtures (natural and RACs) showed decreases in compressive strength, elastic modulus, tensile strength, and mass value, when evaluating the results obtained for samples degraded by 25 and 50 wet-dry cycles.
- The percentage decreases in the properties studied were greater for the normal strength class than for the high-performance class. This evidence is associated with the higher porosity of C35 mixtures.
- For higher number of cycles, it is evident that the capacity of the natural mixture to resist degradation by wetting-drying cycles is greater than RACs, for both C35 and C60. It is believed that this occurs because the natural concrete presents only the fragmentation of the new mortar when subjected to degradation, while RACs undergo this process in both the new mortar and the mortar attached to the RCA particles, suffering, therefore, greater damages.
- A wet-dry degradation law for RAC is proposed in this study for the key mechanical properties after wetting and drying degradation (compressive strength, elastic modulus, and tensile strength) as a function of the initial open porosity of the concrete, which is also directly related to the total mortar volume of concrete.
- Finally, as the RAC resistance to wet-dry degradation is governed by the initial open porosity of the concrete, it would be possible to use RAC in structures subjected to environmental conditions similar to the ones analyzed herein through the control of this physical property in the mix design stage.

## Figures and Tables

**Table 1:** Properties of the natural and recycled aggregates used in this study (adapted from [32]).

Properties	NCA_C0	RCA_L_C0	RCA_D_C0	NCA_C1	RCA_L_C1	RCA_D_C1
D_max_ (mm)	9.5	9.5	9.5	19.0	19.0	19.0
Density (kg/m^3^)	2662	2178	2168	2636	2105	2255
Water Absorption (%)	1.5	7.3	7.6	1.3	8.2	6.1
Attached Mortar (%)	-	44.3	46.2	-	64.8	35.1
Abrasion Wear (%)	39.5	41.2	41.5	36.1	46.7	46.3
Packing Density	0.68/0.54/0.55	0.66/0.60/0.60	0.57/0.55/0.57	0.60/0.56/0.54	0.56/0.55/0.60	0.57/0.57/0.58

**Table 2:** Mixture proportioning of concrete mixtures (adapted from [32]).

Mixtures	w/c_eff_	Materials (kg/m^3^)											Total Mortar (%)
		C1			C0			Sand	CEM	SP	w_eff_	w_tot_	
		NCA	RCA_L	RCA_D	NCA	RCA_L	RCA_D						
C35-NAT	0.60	452	0	0	457	0	0	868	325	1.86	196	212	65.5
C35-L-C0	0.57	451	0	0	0	373	0	866	338	1.93	194	217	74.9
C35-L-C1	0.57	0	361	0	456	0	0	867	336	1.92	191	216	77.6
C35-D-C0	0.55	451	0	0	0	0	371	866	345	1.97	191	214	75.1
C35-D-C1	0.57	0	0	384	453	0	0	862	341	1.95	194	216	73.1
C60-NAT	0.32	448	0	0	452	0	0	860	448	19.20	145	150	65.5
C60-L-C0	0.31	448	0	0	0	371	0	861	458	19.62	141	152	74.8
C60-L-C1	0.30	0	356	0	450	0	0	856	461	19.76	138	151	77.3
C60-D-C0	0.29	448	0	0	0	0	369	860	464	19.89	134	145	74.6
C60-D-C1	0.30	0	0	382	451	0	0	857	463	19.84	137	147	72.7

**Table 3:** Testing procedures on natural and recycled concrete.

Tests	Equipment	Sample	Condition
Slump (NBR NM 67 [42])	Abram Cone	Fresh Concrete	Immediately After Mixing
Water Absorption, Voids, and Specific Gravity (NBR 9778 [43])	Immersion and Boiling of Hardened Concrete	3 Cylindrical 100 × 200 mm	28 Days of Curing
Compressive Strength and Elastic Modulus (NBR 5739 [44])	1000 kN Shimadzu Testing Machine at Axial Displacement of 0.1 mm/min	4 Cylindrical 75 × 150 mm	28 days of curing 0, 25 and 50 wet-dry cycles
Splitting Tensile Strength (NBR 7222 [45])	1000 kN Shimadzu Testing Machine at Axial Displacement of 0.3 mm/min	4 Cylindrical 75 × 150 mm	28 Days of Curing 0, 25 and 50 Wet-Dry Cycles

**Table 4:** Consistency of concrete mixtures and physical and mechanical properties of hardened concrete.

Mixtures	Slump	Water Absorption	Void Index	Specific Gravity	f_c,28_	E_c,28_	f_t,28_
	(mm)	(w_open_) (%)	(%)	(kg/m^3^)	(MPa)	(GPa)	(MPa)
C35-NAT	175	3.0	7.0	2303	34.2	21.3	2.7
C35-L-C0	180	3.6	8.1	2239	35.7	22.1	2.7
C35-L-C1	165	3.9	8.6	2221	35.3	21.2	2.9
C35-D-C0	165	3.7	8.4	2238	34.4	21.7	2.9
C35-D-C1	195	3.5	7.8	2247	33.5	20.9	2.6
C60-NAT	165	1.1	2.7	2411	60.1	29.1	3.9
C60-L-C0	180	1.7	4.0	2354	60.5	29.8	4.0
C60-L-C1	170	1.9	4.3	2339	61.9	30.1	4.4
C60-D-C0	165	1.6	3.8	2361	62.6	31.0	4.4
C60-D-C1	160	1.4	3.3	2376	59.7	29.5	4.1

**Table 5:** Mechanical properties in the wet-dry durability study.

Class	Mixtures	Compressive Strength (MPa)				Elastic Modulus (GPa)				Tensile Strength (MPa)			
		25 WD		50 WD		25 WD		50 WD		25 WD		50 WD	
		Ref	Deg	Ref	Deg	Ref	Deg	Ref	Deg	Ref	Deg	Ref	Deg
C35	C35-NAT	39.1	36.4	39.9	34.1	23.5	22.6	23.9	20.6	2.9	2.7	3.1	2.7
	C35-L-C0	42.1	39.0	43.3	35.9	24.1	22.9	24.6	20.7	3.2	3.0	3.3	2.7
	C35-L-C1	39.9	36.8	41.1	32.9	23.8	22.2	24.3	19.9	3.0	2.7	3.2	2.6
	C35-D-C0	41.1	37.9	42.1	34.3	24.4	22.8	24.8	20.7	3.1	2.9	3.2	2.6
	C35-D-C1	39.6	36.8	41.1	34.1	23.6	22.4	24.2	20.6	2.9	2.7	3.1	2.6
C60	C60-NAT	65.0	62.9	68.2	61.6	32.3	31.4	34.1	30.4	4.1	3.9	4.2	3.8
	C60-L-C0	64.9	62.2	67.9	59.7	33.1	31.7	34.4	29.6	4.2	3.9	4.3	3.7
	C60-L-C1	68.0	64.9	70.3	60.8	32.5	31.0	34.6	29.6	4.5	4.2	4.6	3.9
	C60-D-C0	65.6	62.6	67.1	59.4	32.9	31.9	33.9	29.3	4.5	4.3	4.5	3.9
	C60-D-C1	63.9	61.7	65.8	58.5	32.5	31.5	33.6	29.4	4.3	4.1	4.4	3.9

**Table 6:** Calibrated numerical parameters.

Class	Number of Cycles	Parameter		
		a	b	c
C35	25	0.170 (* c.c. = 0.88)	0.150 (* c.c. = 0.92)	0.195 (* c.c. = 0.87)
	50	0.245 (* c.c. = 0.97)	0.235 (* c.c. = 0.93)	0.245 (* c.c. = 1.00)
C60	25	0.150 (* c.c. = 0.89)	0.125 (* c.c. = 0.87)	0.155 (* c.c. = 0.86)
	50	0.295 (* c.c. = 0.98)	0.305 (* c.c. = 0.99)	0.305 (* c.c. = 0.98)

* c.c. correlation coefficient.
